# Special Issue “Innovations in the Food System: Exploring the Future of Food”

**DOI:** 10.3390/foods11152183

**Published:** 2022-07-22

**Authors:** Maria Lisa Clodoveo

**Affiliations:** Interdisciplinary Department of Medicine, Piazza Giulio Cesare, University of Bari, 70124 Bari, Italy; marialisa.clodoveo@uniba.it

The Food and Agriculture Organization of the United Nations (FAO) in 2018 provided a definition of “food systems” highlighting that they “encompass the entire range of actors and their interlinked value-adding activities involved in the production, aggregation, processing, distribution, consumption, and disposal of food products that originate from agriculture, forestry or fisheries, and food industries, and the broader economic, societal and natural environments in which they are embedded”. The COVID-19 pandemic has imposed a stop that has caused the population to rethink their lifestyles, production, and consumption, also accelerating the transformation progress necessary in light of the objectives of the 2030 Agenda for Sustainable Development—adopted by all United Nations Member States in 2015—which provides a shared blueprint for the peace and prosperity of people and the planet, now and in the future. Actual food systems account for nearly one-third of global GHG emissions; consume large amounts of natural resources; result in biodiversity loss and negative health impacts (due to both under- and over-nutrition); and do not allow fair economic returns and livelihoods for all actors, in particular, for primary producers. With regard to these observations, innovations should aim to develop the following food systems:

Inclusive: ensuring economic and social inclusion for all food system actors, especially smallholders, women, and youth;

Sustainable: minimizing negative environmental impacts, conserving scarce natural resources, and strengthening resiliency against future shocks;

Efficient: producing adequate quantities of food for global needs while minimizing post-harvest loss and consumer waste;

Nutritious and healthy: enabling the consumption of a diverse range of healthy, nutritious, and safe foods.

These are ambitious goals that will require multidisciplinary effort—from engineering to life sciences, biotechnology, medical sciences, social sciences, and economic sciences. New technologies and scientific discoveries are the solution to the increasing demand for sufficient, safe, healthy, and sustainable foods influenced by the increased public awareness of their importance. This Special Issue is composed of 11 papers.

Jing Xu and his collaborators [1] focused their work on strategy optimization of quality improvement and price subsidy of the agri-foods supply chain. In this paper, the differences in the quality safety, price, and market demand of agri-foods in the supply chain are compared and analyzed demonstrating that the maximum profit of supply chain participants decreases with the increase of price elasticity of demand. When the quality of agri-foods is upgraded in a producer-led manner, the quality of agri-foods in the supply chain does not undergo substantial improvement, and the maximum profit of agri-foods operators is insensitive to the price elasticity of demand at this time. When the seller-led quality upgrading is launched, the maximum profit of the producer decreases with the increase of the quality elasticity of demand, the maximum profit of the seller increases with the increase of the quality elasticity of demand, and the total profit of the supply chain also increases with the increase of the quality elasticity of demand under the centralized decision situation. The quality and safety of agri-foods as well as the overall profit of the supply chain can be improved most effectively under the centralized control decision with the goal of maximizing the supply chain benefits. The authors concluded that in terms of quality and price, quality improvement actions of agri-foods driven by supply-side producers are less effective than those driven by demand-side consumption. In addition, cost-sharing contracts can significantly improve the quality of agri-foods in the supply chain and make them more “high-quality and low-price” than before the adoption of cost-sharing contracts.

Giordano Stella and co-authors [2] discussed the “Food Village”, an innovative alternative food network based on a human scale development economic model. The researchers suggest that although the different alternative food networks (AFNs) have experienced increases worldwide for the last thirty years, they are still unable to provide an alternative capable of spreading on a large scale. They in fact remain niche experiments due to some limitations on their structure and governance. Their study proposes and applies a design method to build a new sustainable food supply chain model capable of realizing a “jumping scale”. Based on the theoretical and value framework of the Civil Economy (CE), the Economy for the Common Good (ECG), and the Development on a Human Scale (H-SD), the proposed design model aims to satisfy the needs of all stakeholders in the supply chain. Max-Neef’s Needs Matrix and Design Thinking (DT) tools were used to develop the design model. Applying the design method to the food chain has allowed us to develop the concept of the “Food Village”, an innovative food supply network far from the current economic mechanisms and based on the community and eco-sustainability.

Maiorano et al. [3] addressed the ambiental issue of Food from the Depths of the Mediterranean and the role of habitats, changes in the sea-bottom temperature, and fishing pressure. This paper reviews studies that highlight a link between deep-sea fishery resources (deep-sea food resources) and vulnerable marine ecosystems, species, and habitats in the Mediterranean Sea, providing new insights into changes in commercial and experimental catches of the deep-sea fishery resources in the central Mediterranean over the last 30 years. About 40% of the total landing of Mediterranean deep-water species is caught in the central basin. Significant changes in the abundance of some of these resources with time, sea-bottom temperature, and fishing effort have been detected, as well as an effect of the Santa Maria di Leuca cold-water coral province on the abundance of the deep-sea commercial crustaceans and fishes. The implications of these findings and the presence of several geomorphological features, sensitive habitats, and vulnerable marine ecosystems in the central Mediterranean are discussed with respect to the objectives of biodiversity conservation combined with those of management of fishery resources.

Estee Ngew and her research team [4] studied the composite of layered double hydroxide with casein and carboxymethylcellulose as a white pigment for food application. The authors studied the composite of layered double hydroxide with casein and carboxymethylcellulose as an alternative to the titanium dioxide (TiO_2_). Titanium dioxide is commonly used in food, cosmetic, and pharmaceutical industries as a white pigment due to its extraordinary light scattering properties and high refractive index. However, as evidenced by recent reports, there are overriding concerns about the safety of nanoparticles of TiO_2_. As an alternative to TiO_2_, Mg-Al layered double hydroxide (LDH) and their composite containing casein and carboxymethyl cellulose (CMC) were synthesized using wet chemistry and compared with currently used materials (food grade TiO_2_ (E171), rice starch, and silicon dioxide (E551)) for its potential application as a white pigment. These particles were characterized for their size and shape (Transmission Electron Microscopy), crystallographic structure (X-ray Diffraction), agglomeration behavior and surface charge (Dynamic Light Scattering), surface chemistry (Fourier Transform Infrared Spectroscopy), transmittance (UV–VIS spectroscopy), masking power, and cytotoxicity. their results showed the formation of typical layered double hydroxide with flower-like morphology which was restructured into pseudo-spheres after casein intercalation. Transmittance measurement showed that LDH composites had better performance than pristine LDH, and the aqueous suspension was heat and pH-resistant. While its masking power was not on a par with E171, the composite of LDH was superior to current alternatives such as rice starch and E551. Sustainability score obtained by MATLAB^®^ based comparison for price, safety, and performance showed that LDH composite was better than any of the compared materials, highlighting its potential as a white pigment for applications in food.

Caterina Palocci and collaborators [5] presented their preliminary results on a search engine concept to improve food traceability and transparency. The authors started from the evidence that in recent years, the digital revolution has involved the agrifood sector. However, the use of the most recent technologies is still limited due to poor data management. The integration, organization, and optimized use of smart data provide the basis for intelligent systems, services, solutions, and applications for the food chain management. With the purpose of integrating data on food quality, safety, traceability, transparency, and authenticity, an EOSC-compatible (European Open Science Cloud) traceability search engine concept for data standardization, interoperability, knowledge extraction, and data reuse, was developed within the framework of the FNS-Cloud project (GA No. 863059). For the developed model, three specific food supply chains were examined (olive oil, milk, and fishery products) in order to collect, integrate, organize, and make available data relating to each step of each chain. For every step of each chain, parameters of interest and parameters of influence—related to nutritional quality, food safety, transparency, and authenticity—were identified together with their monitoring systems. The developed model can be very useful for all actors involved in the food supply chain, both to have a quick graphical visualization of the entire supply chain and for searching, finding, and re-using available food data and information.

Gianfranco Spizzirri [6] and its research group developed a tara gum/olive mill wastewaters phytochemicals conjugate as a new ingredient for the formulation of an antioxidant-enriched pudding. Olive mill wastewater, a high polyphenols agro-food by-product, was successfully exploited in an eco-friendly radical process to synthesize an antioxidant macromolecule, usefully engaged as a functional ingredient to prepare functional puddings. The chemical composition of lyophilized olive mill wastewaters was investigated by HPLC-MS/MS and 1H-NMR analyses, while the antioxidant profile was in vitro evaluated by colorimetric assays. Oleuropein aglycone (5.8 μg mL^−1^) appeared as the main compound, although relevant amounts of an isomer of the 3-hydroxytyrosol glucoside (4.3 μg mL^−1^) and quinic acid (4.1 μg mL^−1^) were also detected. LOMW was able to greatly inhibit ABTS radical (IC50 equal to 0.019 mg mL^−1^), displaying, in the aqueous medium, an increase in its scavenger properties by almost one order of magnitude compared to the organic one. Lyophilized olive mill wastewaters reactive species and tara gum chains were involved in an eco-friendly grafting reaction to synthesize a polymeric conjugate that was characterized by spectroscopic, calorimetric, and toxicity studies. In vitro acute oral toxicity was tested against 3T3 fibroblasts and Caco-2 cells, confirming that the polymers do not have any effect on cell viability at the dietary use concentrations. Antioxidant properties of the polymeric conjugate were also evaluated, suggesting its employment as a thickening agent, in the preparation of pear puree-based pudding. High performance of consistency and relevant antioxidant features over time (28 days) were detected in the milk-based foodstuff, in comparison with its non-functional counterparts, confirming lyophilized olive mill wastewaters as an attractive source to achieve high-performing functional foods.

Vincenzo Russo et al. [7] studied the role of territoriality and safety perception on the intention to buy dairy products with certification marks. Over the years, the territorial origins of agri-food products have become a consolidated marketing model which stands as an alternative to mass production. References to territory, whether on the packaging or in advertising, have become an increasingly popular way for marketers to differentiate products, by attributing specific characteristics to them, derived from specific cultural identities and traditions. The aim of their study was to capture the possible differences between two groups, Italian and French, in the perception and intention to buy products with certification marks. The authors tested a multi-group structural equations model, assessing the mediation of the Perceived Product Safety between Packaging with reference to Territoriality and Intention to Buy. The authors’ findings show that in both groups Packaging with reference to Territoriality has a positive association with Intention to Buy and Perceived Product Safety and that Perceived Product Safety has a positive association with Intention to Buy. The difference is the mediation of Perceived Product Safety, present only in the Italian group. This opens important considerations on the role of the perception of safety, particularly in the pandemic period, in the presentation of products, particularly in products with certification marks linked to sustainability and territoriality.

Tao et al. [8] wrote an article on Big Data in Food Industry. The authors underlined that a huge amount of data is being produced in the food industry, but the application of big data—regulatory, food enterprise, and food-related media data—is still in its infancy. Each data source has the potential to develop the food industry, and big data has broad application prospects in areas such as social co-governance, exploit of consumption markets, quantitative production, new dishes, take-out services, precise nutrition, and health management. However, there are urgent problems in technology, health, and sustainable development that need to be solved to enable the application of big data to the food industry. The results showed the great potential for big data in the food industry. Big data has particularly broad application prospects in social co-governance of the food industry, quantitative production, exploitation of consumption markets, new dishes, take-out services, and precise nutrition and health management.

Simone Mancini with the research team [9] discussed the future of edible insects in Europe. They started from the evidence that the effects of population increase and food production on the environment have prompted various international organizations to focus on the future potential for more environmentally friendly and alternative protein products. One of those alternatives might be edible insects. Entomophagy, the practice of eating insects by humans, is common in some places but has traditionally been shunned in others, such as European countries. The last decade has seen a growing interest from the public and private sectors in the research in the sphere of edible insects, as well as significant steps forward from the legislative perspective. In the EU, edible insects are considered novel foods, therefore a specific request and procedure must be followed to place them on the market; in fact, until now, four requests regarding insects as a novel food have been approved. Insects could also be used as feed for livestock, helping to increase food production without burdening the environment (indirect entomophagy). Market perspectives for the middle of this decade indicate that most of the demand will be from the feed sector (as pet food or livestock feed production). Undoubtedly, this sector is gaining momentum and its potential relies not only on food, but also on feed in the context of a circular economy.

Clodoveo and her collaborators [10] presented an overview of innovative packaging methods aimed to increase the shelf-life of cook-chill foods. Analyzing the changing citizen habits, it is clear that the consumption of meals prepared, packaged, and consumed inside and outside the home is increasing globally. This is a result of rapid changes in lifestyles as well as innovations in advanced food technologies that have enabled the food industry to produce more sustainable and healthy fresh packaged convenience foods. This paper presents an overview of the technologies and compatible packaging systems that are designed to increase the shelf-life of foods prepared by cook–chill technologies. The concept of shelf-life is discussed and techniques to increase the shelf life of products are presented including active packaging strategies.

The last paper of this collection has been written by Clodoveo et al. [11]. and is entitled “The Tower of Babel of Pharma-Food Study on Extra Virgin Olive Oil Polyphenols”. Much research has been conducted to reveal the functional properties of extra virgin olive oil polyphenols on human health once extra virgin olive oil is consumed regularly as part of a balanced diet, as in the Mediterranean lifestyle. Despite the huge variety of research conducted, only one effect of extra virgin olive oil polyphenols has been formally approved by EFSA as a health claim. This is probably because EFSA’s scientific opinion is entrusted to scientific expertise about food and medical sciences, which adopt very different investigative methods and experimental languages, generating a gap in the scientific communication that is essential for the enhancement of the potentially useful effects of extra virgin olive oil polyphenols on health. Through the model of the Tower of Babel, we propose a challenge for science communication, capable of disrupting the barriers between different scientific areas and building bridges through transparent data analysis from the different investigative methodologies at each stage of health benefits assessment. The goal of this work is the strategic, distinctive, and cost-effective integration of interdisciplinary experiences and technologies into a highly harmonious workflow, organized to build a factual understanding that translates, because of trade, into health benefits for buyers, promoting extra virgin olive oils as having certified health benefits, not just as condiments.

As summarized above, the collection of 11 articles that make up this Special Issue devoted to “Innovations in the Food System: Exploring the Future of Food” underscores the progress that has been made toward a better understanding of the trends in the global food industry, oriented to a sustainable production assisted by economic and technological tools. surely these first evidences will be developed and enriched in the near future and additional studies are expected to cover a broad range of approaches to the design of innovation in the Food systems.
